# Investigating the human protein-host protein interactome of SARS-CoV-2 infection in the small intestine 

**Published:** 2020

**Authors:** Mahmoud Khodadoost, Zahra Niknam, Masoumeh Farahani, Mohammadreza Razzaghi, Mohsen Norouzinia

**Affiliations:** 1 *Department of Traditional Medicine, School of Traditional Medicine, Shahid Beheshti University of Medical Sciences, Tehran, Iran *; 2 *Proteomics Research Center, Shahid Beheshti University of Medical Sciences, Tehran, Iran *; 3 *Laser Application in Medical Sciences Research Center, Shahid Beheshti University of Medical Sciences, Tehran, Iran *; 4 *Gastroenterology and Liver Diseases Research Center, Research Institute of Gastroenterology and Liver Diseases, Shahid Beheshti University of Medical Sciences, Tehran, Iran *

**Keywords:** SARS-CoV-2, Small intestine, Interactome, Protein interaction network, Regulatory network, Drug targets

## Abstract

**Aim:**

The present study aimed to identify human protein–host protein interactions of SARS-CoV-2 infection in the small intestine to discern the potential mechanisms and gain insights into the associated biomarkers and treatment strategies.

**Background:**

Deciphering the tissue and organ interactions of the SARS-CoV-2 infection can be important to discern the potential underlying mechanisms. In the present study, we investigated the human protein–host protein interactions in the small intestine.

**Methods:**

Public databases and published works were used to collect data related to small intestine tissue and SARS-CoV-2 infection. We constructed a human protein-protein interaction (PPI) network and showed interactions of host proteins in the small intestine. Associated modules, biological processes, functional pathways, regulatory transcription factors, disease ontology categories, and possible drug candidates for therapeutic targets were identified.

**Results:**

Thirteen primary protein neighbors were found for the SARS-CoV-2 receptor ACE2. ACE2 and its four partners were observed in a highly clustered module; moreover, 8 host proteins belonged to this module. The protein digestion and absorption as a significant pathway was highlighted with enriched genes of ACE2, MEP1A, MEP1B, DPP4, and XPNPEP2. The HNF4A, HNF1A, and HNF1B transcription factors were found to be regulating the expression of ACE2. A significant association with 12 diseases was deciphered and 116 drug-target interactions were identified.

**Conclusion:**

The protein-host protein interactome revealed the important elements and interactions for SARS-CoV-2 infection in the small intestine, which can be useful in clarifying the mechanisms of gastrointestinal symptoms and inflammation. The results suggest that antiviral targeting of these interactions may improve the condition of COVID-19 patients.

## Introduction

 COVID-19 disease as a new type of beta-coronavirus disease first emerged in Wuhan, China during December 2019, and is currently spreading rapidly throughout the world. The 2019-nCOV has caused cases of COVID-19 disease throughout the world today (1). Until August 24, 2020, a total of 23.4 million COVID-19 cases have been diagnosed, with 809,000 deaths worldwide. The World Health Organization (WHO) has identified this disease as a global health threat and has supported every research in finding a vaccine (2). The major way of cause infection is human-to-human transmission (3), and despite the rapid spread of this virus, the detailed molecular mechanism and pathogenesis of it remain unclear. The main clinical symptoms of COVID-19 cases are fever, cough, and sore throat (4), however, gastrointestinal symptoms appear in some patients (2%-10%) especially in younger COVID-19 patients, including diarrhea, vomiting, and abdominal pain, proposing that not only does COVID-19 involve lung tissue but that the digestive system can also be affected by SARS-CoV-2 (5, 6). The almost 86.9% similarity of the nucleotide sequence of SARS-COV2 to the SARS-COV and also the pairwise protein sequence analysis results suggest that COVID-19 uses a similar mechanism to enter the host cell (7, 8). In addition, previous studies suggested that angiotensin-converting enzyme 2 (ACE2) is the major receptor of SARS-COV; playing an important role in the entry of the SARS-CoV-2 virus into the host cell (9). So, it can be concluded that ACE2 can be a potential vaccine target for COVID-19 disease therapy. ACE2 greatly express in human organs including lung epithelial cells, kidney, heart, blood, and the small intestine (10). The widespread expression of ACE2 in different tissues might indicate the multi-organ dysfunction in COVID-19 patients (11). Interestingly, the expression of ACE2 protein is downregulated after host infection with SARS-COV (12). Decreased ACE2 expression levels can increase susceptibility to intestinal inflammation due to damage to epithelial cells and its expression on surface cells of the small intestine might participate in the virus invasion, amplification, and also activation of gastrointestinal inflammation (12). Due to the genomic similarity of SARS-CoV-2 to SARS-CoV, SARS-CoV-2 may also impair ACE2 expression, and this reduction or inhibition of ACE2 expression can lead to dysfunction in the gastrointestinal tract, resulting in digestive symptoms in COVID-19 patients, and can also be a possible molecular pathway for adverse digestive symptoms emerging in patients with COVID-19. On the other hand, ACE2 also plays a key role in the intestine including maintaining amino acid homeostasis (13). Constant expression of ACE2 on the small intestine may relate to gastrointestinal symptoms, impact the infection route, and virus pathogenicity (11). So far, there are no effective antiviral drugs or vaccines against SARS-CoV-2, which is due to the limited understanding of the molecular pathways of coronavirus pathogenesis (14). Understanding COVID-19 disease pathogenesis and also how the COVID-19 causes gastrointestinal symptoms in our body are crucial points to be considered (15), and the detection of host protein interactions with ACE2 in the small intestine could help to discover coronavirus pathology pathways and present potential drug and vaccine targets. The viral entry can affect the interaction of the ACE2 with host cellular proteins. Accordingly, protein interaction analyses may provide further insight into understanding the molecular basis of COVID-19 pathogenesis. Besides, identification and knowledge of the distribution of ACE2 receptor in various human tissues are of particular importance in discovering treatment strategies for COVID-19 infection. Small intestine enterocytes show a high expression of ACE2 mRNA and protein (16, 17). In this study, to provide a view into the biological processes and pathways in the host cell by COVID-19 infection, and gastrointestinal inflammation, we analyzed the human protein-host protein interactome of the SARS-CoV-2 infection in the small intestine using protein interaction and a transcription factor regulatory network. Furthermore, we extracted possible drug-target interactions. 

## Methods

This study aimed to identify possible human protein–host protein interactions of SARS-CoV-2 infection in the small intestine and attempted to discern the potential mechanisms of gastrointestinal symptoms in the COVID-19 disease.

**Data related to small intestine tissue and SARS-CoV-2 infection**

The small intestine-specific genes list was retrieved from the Tissue-specific Gene Expression and Regulation (TiGER) database (18). Various recent studies have presented interaction networks of ACE2 (as a major host receptor for SARS-CoV-2) and some reports have exhibited specific expression of it in small intestinal enterocytes after virus infection (17, 19-21). Hence, in this study, the first interacting protein neighbors of ACE2 were gathered from the UniProt (22) and GeneCards databases (23) based on the interaction records in the STRING database (24). The serine protease TMPRSS2 as a host factor that is required for SARS-CoV-2 entry (25) was also added to the list. The human host proteins of SARS-CoV-2 were compiled from two different studies by Gordon et al. and Kumar et al (26, 27). After obtaining these lists of proteins, the tissue specific- expression analysis of the protein list was performed by the Database for Annotation, Visualization and Integrated Discovery (DAVID) webserver using the “UNIGENE_EST_QUARTILE” categories (28). The signiﬁcant tissue-specific genes for the small intestine were selected with an FDR corrected p-value < 0.05. The resultant gene list was used for further analysis.

**Figure 1:**
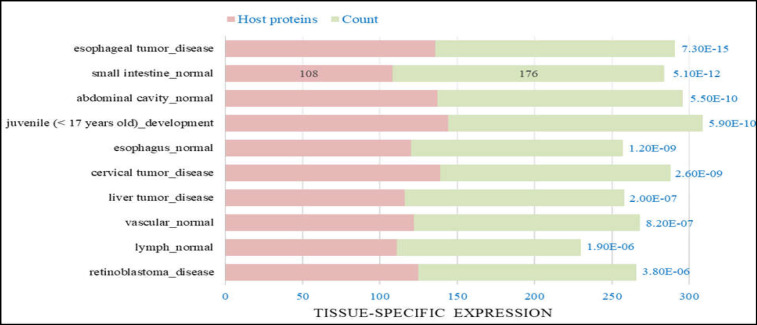
Top 10 tissue-specific expression categories with FDR-corrected p-value <. 05. 176 genes for small intestine tissue were enriched with FDR-corrected p-value = 5.10E-12, of which 108 genes were common with the host protein list

**Network generation, modules detection, and functional analyses**

Small intestine tissue-specific genes were queried into the STRING database to mine the related protein-protein interactions (PPI) (24). STRING is a web resource for exploiting the known and predicted PPI information. The combined score > 0.4 was considered as a criterion for the selection of protein interactions. These connections were then imported into the Cytoscape software to build a PPI network (29). The host proteins were determined using a different color in the resulting network. ACE2 interactome network, in the resultant network of small intestine tissue, was presented using ACE2, the first interacting protein neighbors of ACE2, and second neighbor host proteins. A module is a cluster of highly interacting proteins. The molecular complex detection (MCODE) algorithm was employed to screen the high modularity clusters of the PPI network in the Cytoscape software (30). Moreover, cross-talk and interactions between the detected modules and ACE2 were extracted. Functional roles of the high score clusters were evaluated through gene ontology biological processes enrichment, and KEGG (Kyoto Encyclopedia of Genes and Genomes) (31) pathway analysis, with the aid of the DAVID database (28). 

**Regulatory network construction**

Possible upstream regulating transcription factors of the 176 small intestine-specific proteins were enriched using the GeneTrail2 web service (32). GeneTrail2 as a web-interface provides access to different tools for biological analyses and extraction of molecular signatures. The validated transcription factor-target gene interactions with q-value < 0.05 were retrieved from the TRANSFAC database through the GeneTrail2 web service (33). The transcription factor-target gene interactions were imported into Cytoscape to generate the regulatory network. 

**Disease Ontology Annotation and disease-gene network construction**

Overlapping genes across the 176 genes of small intestine tissue and different diseases were mined using the GAD (Genetic Association Database)_DISEASE category with the aid of the DAVID database (28). P-value < 0.05 was considered a specifying parameter for the identification of GAD_DISEASE ontology terms (34). Disease-gene associations were imported into Cytoscape to generate a disease-gene network. 

**Prediction of drug-target interactions and generation of drug-protein interaction network**

Drugs for the enriched KEGG pathways were obtained from the KEGG database (31). Moreover, overlapping diseases with the 176 genes list were queried in repoDB drug repositioning database (35). The resultant drugs list from the KEGG and repoDB databases with the 176 genes list were merged and compiled into one list and mapped into the STITCH database to identify possible drug-target interactions (36). The drug-target interactions were imported into Cytoscape to construct a drug-protein interaction network. 

## Results

**Data preparation for analysis**

A list of 101 small intestine-specific genes was retrieved from TiGER. ACE2, 17 proteins as the first interacting protein neighbors of ACE2, and TMPRSS2 were added to the gene list. Lists of 508 host proteins were compiled from studies of Gordon et al. (332 host proteins) and Kumar et al. (390 host proteins) (26, 27). Finally, a comprehensive list of 625 genes was compiled. Subsequently, the gene list was queried to the DAVID database to identify tissue-specific expression categories, which showed 176 significant small intestine-specific genes (108 host proteins) with FDR-corrected p-value = 5.10E-12 as shown in Figure 1. The 176 genes list was used to construct a PPI network.

**Network generation, modules detection, and functional analyses**

**Figure 2.**
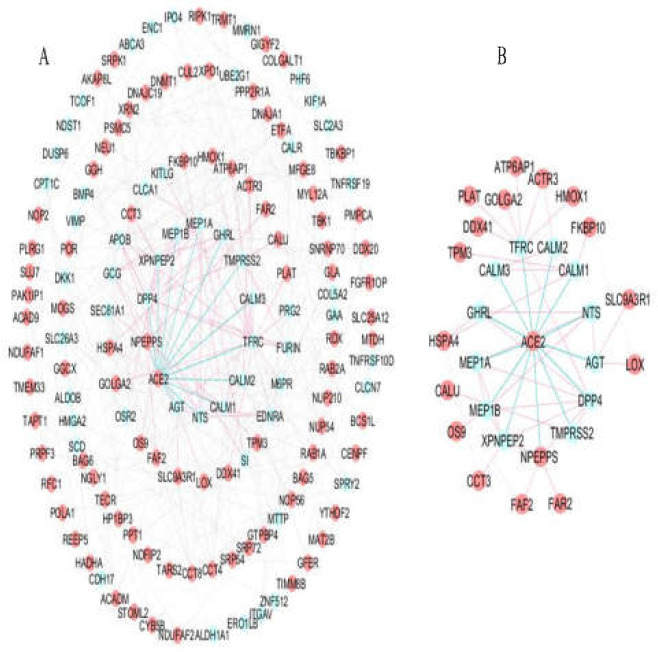
(A). PPI Network of the 176 small intestine-specific proteins (146 nodes and 366 edges). Red nodes: host proteins (91 proteins). Blue edges: ACE2-first neighbor interactions. Red edges: ACE2-second neighbor interactions. (B). ACE2 interaction network in the small intestine including ACE2, the first interacting protein neighbors of ACE2 (13 proteins), and second neighbor host proteins (16 proteins)

**Figure 3:**
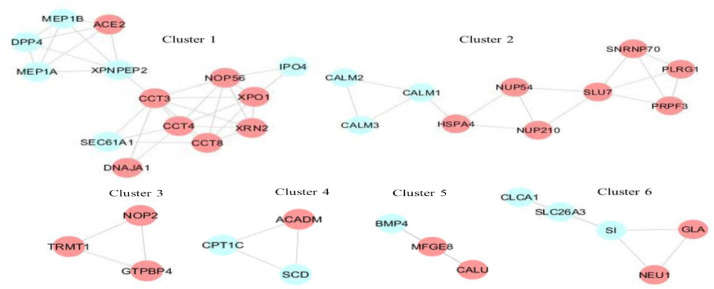
Skeletal structure of 6 clusters. Red nodes: host proteins

**Figure 4:**
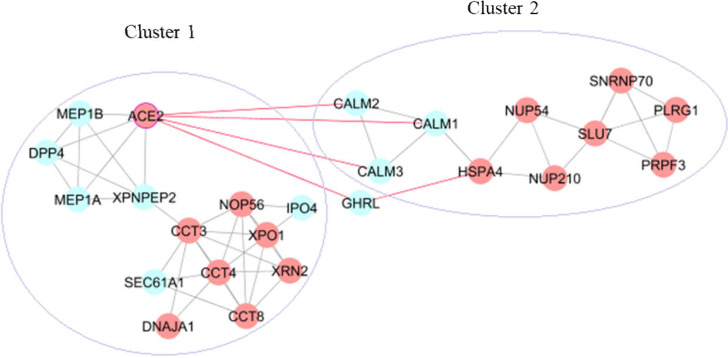
Cross-talk among the clusters through ACE2 in the network. Red nodes: host proteins

Human protein interactions with a medium conﬁdence score for the 176 small intestine-specific genes were mined from the STRING database. These associations were imported into Cytoscape and a scale-free PPI network with an r-squared value of 0.764 was constructed that showed 146 nodes (91 host proteins) and 366 edges (Figure 2. (A)). Thirteen interactions were found for ACE2 with small intestine-specific proteins. Figure 2 (B) presents possible ACE2 interactome in the small intestine including ACE2, the first interacting protein neighbors of ACE2 (DPP4, MEP1A, MEP1B, AGT, NTS, TFRC, GHRL, NPEPPS, TMPRSS2, CALM1, CALM2, CALM3, and XPNPEP2), and the second interacting host protein neighbors (PLAT, ACTR3, HSPA4, OS9, CCT3, FAR2, CALU, SLC9A3R1, LOX, FKBP10, FAF2, ATP6AP1, HMOX1, DOX41, TPM3, and GOLGA2). Six modules were detected with MCODE scores > 3. Figure 3 demonstrates the skeletal structure of the 6 clusters. The first cluster with the highest MCODE score of 5.077 showed 14 nodes and 33 edges. ACE2 and 4 partners of ACE2 (DPP4, MEP1A, MEP1B, and XPNPEP2) were seen in Cluster1. Moreover, 8 host proteins (ACE2, XPO1, DNAJA1, XRN2, CCT3, CCT8, CCT4, and NOP56) belonged to Cluster1. The results revealed Cluster2 with an MCODE score of 3.333, 10 nodes (7 host proteins), and 15 edges. Cross-talk among Cluster1 and other clusters through ACE2 was investigated that showed a direct interaction of ACE2 with CALM1, CALM2, and CALM3 in Cluster2. An indirect interaction was also seen through GHRL that connects ACE2 in Cluster1 to the HSPA4 host protein in Cluster2 (Figure 4). Functional analysis of Cluster1 enriched the protein digestion and absorption KEGG pathway with FDR corrected p-value < 0.05. 

ACE2 and MEP1B, MEP1A, DPP4, XPNPEP2 proteins were significantly enriched in this KEGG pathway (Table 1). Additionally, gene ontology (GO) biological processes terms were detected, for example, toxin transport, regulation of protein localization to Cajal body, and protein localization to nuclear body. 

**Table 1:** Gene ontology and KEGG pathway analysis of Cluster1

Category	Term	FDR	Genes
KEGG_PATHWAY	protein digestion and absorption	0.0018	MEP1A, ACE2, MEP1B, DPP4, XPNPEP2
GOTERM_BP_ALL	toxin transport	2.36E-05	CCT4, CCT8, DNAJA1, MEP1B, CCT3
GOTERM_BP_ALL	regulation of protein localization to Cajal body	0.0232	CCT4, CCT8, CCT3
GOTERM_BP_ALL	positive regulation of protein localization to Cajal body	0.0232	CCT4, CCT8, CCT3
GOTERM_BP_ALL	protein localization to Cajal body	0.0298	CCT4, CCT8, CCT3
GOTERM_BP_ALL	protein localization to nuclear body	0.0298	CCT4, CCT8, CCT3

**Regulatory network construction**

**Figure 5:**
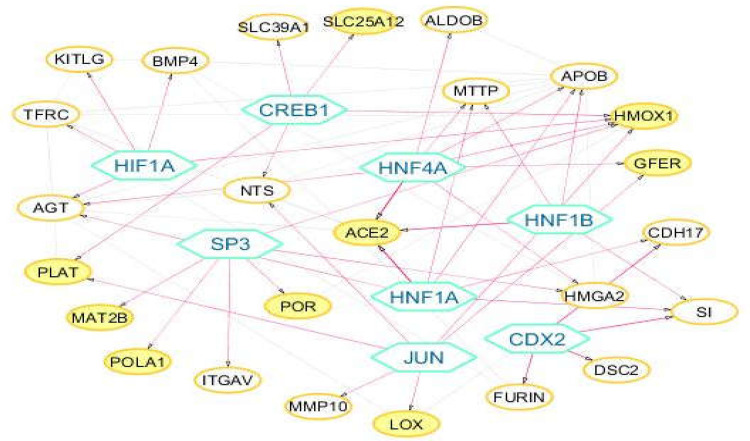
Transcription factor-target gene regulatory network. Hexagon nodes represent the 8 enriched transcription factors. Yellow nodes: host proteins

Eight transcription factors (TFs) (HNF4A, HNF1A, HNF1B, CDX2, JUN, HIF1A, CREB1, and SP3) with q-value < 0.05 were enriched for 25 target genes from the 176 genes list. The regulatory network showed 45 validated transcription factor-target gene interactions between 8 TFs and 25 target genes (Figure 5). HNF factors including HNF4A, HNF1A, and HNF1B were found to be regulating the expression of the ACE2 target gene (Table 2). 

**Disease-gene network construction**

**Table 2:** Enriched transcription factors for the 176 genes list

N	Transcription factor	q-value	Target genes (of the 176 small intestine-specific proteins)
1	HNF4A	9.66E-07	ACE2, AGT, ALDOB, APOB, GFER, HMGA2, HMOX1, MTTP
2	HNF1A	3.14E-05	ACE2, AGT, APOB, CDH17, MTTP, SI
3	HNF1B	1.13E-04	ACE2, APOB, MTTP, SI
4	CDX2	0.0018	CDH17, DSC2, FURIN, SI
5	JUN	0.0027	GFER, HMOX1, LOX, MMP10, NTS, PLAT
6	HIF1A	0.0092	AGT, BMP4, HMOX1, KITLG, TFRC
7	CREB1	0.0208	HMOX1, NTS, PLAT, SLC25A12, SLC39A1
8	SP3	0.0365	HMGA2, HMOX1, ITGAV, MAT2B, POLA1, POR

**Table 3:** Disease ontology of 176 genes

Category	Term	Count	P-Value
GAD_DISEASE	Type 2 Diabetes| edema | rosiglitazone	45	7.60E-06
GAD_DISEASE	blood pressure, arterial	5	2.80E-03
GAD_DISEASE	Acquired Immunodeficiency Syndrome|Disease Progression	20	3.00E-03
GAD_DISEASE	Cardiovascular Diseases|	5	5.30E-03
GAD_DISEASE	Cleft Lip|Cleft Palate	11	8.70E-03
GAD_DISEASE	Hyperlipidemias|Hypertension	3	1.00E-02
GAD_DISEASE	Spinal Dysraphism	4	2.50E-02
GAD_DISEASE	protein C protein S	2	3.10E-02
GAD_DISEASE	restenosis	4	3.20E-02
GAD_DISEASE	longevity	10	3.50E-02
GAD_DISEASE	Colorectal Cancer	10	3.50E-02
GAD_DISEASE	nephropathy, diabetic	3	4.70E-02

**Figure 6:**
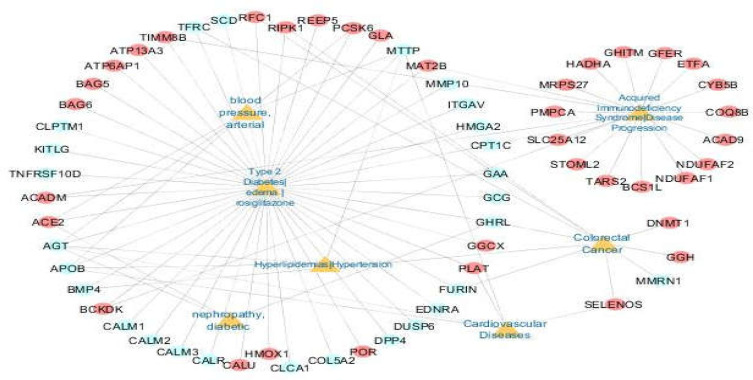
Disease-gene network. Yellow nodes represent the 7 overlapping diseases with 176 genes. Red nodes: host proteins

GAD disease category associations were scanned to further classify the 176 genes and annotating their function. Twelve GAD_DISEASE categories were found with a p-value < 0.05 (Table 3). A disease-gene network was constructed using 7 GAD_DISEASE categories that have highlighted their importance in the COVID-19 response (diabetes, immunodeficiency, cardiovascular diseases, hypertension, blood pressure, colorectal cancer, and nephropathy (Figure 6). Seven GAD_DISEASE categories, 64 genes, and 91 associated-gene interactions are presented in the disease-gene network. Subsequently, these overlapping diseases were queried in repoDB to identify drug candidates. 

**Drug-protein interaction network**

 A list was prepared of 24 drugs that are in the KEGG database for protein digestion and absorption KEGG pathway (the most important pathway was found in our analysis approach). 

**Table 4:** This table presents the drugs list and associated drug classes

Drug classes	Drug names	Drug targets
ACE inhibitors	benazepril, spirapril, trandolapril, cilazapril, fosinopril, moexipril, quinapril	ACE2
	captopril	ACE2, AGT, MOGS
	enalapril, enalaprilat, perindopril, lisinopril	ACE2, AGT
	ramipril	ACE2, HMOX1
Gliptins	anagliptin, carmegliptin, denagliptin, evogliptin, gosogliptin, linagliptin, omarigliptin, saxagliptin, sitagliptin, teneligliptin, trelagliptin, vildagliptin	DPP4
Statins	atorvastatin	ACE2, APOB, BCKDK, DPP4, HMOX1
	simvastatin	APOB, HMOX1, PLAT
Calcium channel blockers	carvedilol	AGT, ETFA
	felodipine, nicardipine, nifedipine	CALM1, CALM2, CALM3
	verapamil	CALM1, CALM2
Angiotensin receptor blockers (ARBs)	valsartan	ACE2, AGT, EDNRA, HMOX1
	irbesartan, losartan	ACE2, AGT, EDNRA
	telmisartan, candesartan, olmesartan	AGT
	olmesartan, hydrochlorothiazide	ACE2
Alpha-blockers	doxazosin	EPHA7
	prazosin, terazosin	EDNRA
	bupranolol	DDX41
Aldosterone receptor antagonists	spironolactone	PLAT
Intravenous anesthetic	propofol	AGT, PLAT, HMOX1
Vasodilators	nitroglycerin	ALDH1A1, PLAT
	hydralazine	AGT, DNMT1
Adrenergic blocking agent	reserpine	GFER
Antibiotic	doxycycline	MMP10, HSPA4
Estrogens	estradiol	ALDH1A1, APOB, BMP4, DNMT1, HMOX1, PLAT
Fatty acid, Omega-6 fatty acid	gamma-linolenic	HMOX1
Antihyperglycemic agent	metformin	DPP4, HMOX1, PLAT
non-steroidal anti-inflammatory drug	aspirin (acetylsalicylic acid)	EDNRA, HMOX1, PLAT
	salicylate	SLC26A3
Monoamine Oxidase Inhibitors	pargyline	ALDH1A1
potassium-sparing diuretic family	amiloride	TMPRSS2, NDFIP2, PLAT
renin inhibitor	aliskiren	ACE2, AGT
Chemotherapy	paclitaxel	FURIN, DNAJA1, KIF1A
Thiazide diuretics	hydrochlorothiazide	ACE2
Retinoids	retinoic acid	ALDH1A1, APOB, BMP4, KITLG, PLAT, TFRC
Androgens	testosterone	ALDH1A1, GHRL, HSPA4, TMPRSS2

**Figure 7:**
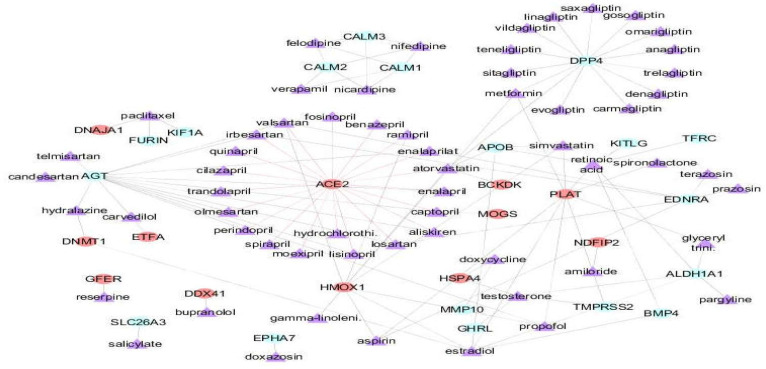
Drug-protein interaction network. Violet nodes represent the 60 drugs. Red nodes: host proteins

Additionally, 110 drugs were mined from repoDB for overlapping diseases with the 176 genes. Subsequently, the resultant drugs along with the 176 genes list were mapped into the STITCH database. Three new drugs were also revealed in the data list that was predicted by STITCH, including gamma-linolenic acid, retinoic acid, and glyceryl trinitrate (nitroglycerin). A Drug-protein interaction network was constructed using the STITCH outcomes in Cytoscape that revealed 116 drug-protein interactions, including 30 proteins (12 host proteins) and 60 drugs. The drug candidates mostly belong to the drug classes of ACE inhibitors, Angiotensin receptor blockers (ARBs), calcium channel blockers, gliptins, and statins (Figure 7, Table 4). ACE2 showed 20 drug interactions.

## Discussion

As recent studies have suggested, SARS-CoV-2 may infect the host cells by the virus binding to the ACE2 receptor. It could be assumed, then, that ACE2 may be a potential therapeutic target against COVID-19 (37). Previous studies have reported the expression pattern of the ACE2 gene as a host cell receptor of SARS-CoV-2 in the small intestine (17). They have demonstrated ACE2 expression on the surface enterocytes that potently facilitates the entrance of SARS-CoV-2 and results in host infection and gastrointestinal symptoms (17). In this study, we aimed to identify the SARS-CoV-2–host protein interactions in the small intestine and understand the possible mechanisms of gastrointestinal symptoms in the COVID-19 disease to gain insights about the associated biomarkers. PPI network of the small intestine-specific proteins revealed the 13 ACE2 interacting partners (DPP4, MEP1A, MEP1B, AGT, NTS, TFRC, GHRL, NPEPPS, TMPRSS2, CALM1, CALM2, CALM3, and XPNPEP2) and their associated functional pathways in the small intestine. ACE2 and four of its primary protein neighbors (DPP4, MEP1A, MEP1B, and XPNPEP2) were seen in the highly clustered module enriched in the protein digestion and absorption KEGG pathway. ACE2 is abundantly presented on the luminal surface of intestinal epithelial cells and functions as a co-receptor for amino acid and nutrient uptake from food (38). The deficiency of ACE2 in the intestine, due to SARS-CoV-2 infection, may further impair the nutrition of the host and have abilities to mount a balanced immune response (39). Moreover, several studies have demonstrated that ACE2 also functions as the chaperone for the membrane trafficking of the amino acid transporter B^0^AT1 (40). Therefore, when SARS-CoV-2 blocks ACE2 it also blocks B^0^AT1, thus shutting down intestinal amino acid transport (40). It was shown that mutations in B^0^AT1 led to Hartnup disease with defective adsorption of amino acids through the kidneys and small intestine. Patients with Hartnup disease develop pellagra-like symptoms under stress conditions, such as rash, cerebellar ataxia, and diarrhea (39). In ACE2 deficient mice, B^0^AT1 is completely absent from the small intestine and, therefore, displays a dramatically decreased level of tryptophan which also causes colitis (13, 40). Since tryptophan-rich peptides are a subset of antimicrobial peptides, the interaction of SARS-CoV-2 with the enterocytic ACE2/B^0^AT1 receptors can cause an abnormal composition of the gut microbiome followed by a massive inflammatory response and cytokine storm in the small bowel (39). Meprins (MEP1A, MEP1B), as shown in the results, are the first interacting protein neighbors of ACE2. They are extracellular proteases involved in connective tissue homeostasis, intestinal barrier function, and immunological processes (41). MEP1A has been identified as a genetic susceptibility factor for inflammatory bowel disease. It encodes meprin α, an astacin-like metalloprotease, which is secreted into the intestinal lumen or it is preserved at the brush border membrane in relation to transmembrane meprin β (42). Therefore, any reduction in meprin α or β expression can cause similar defects in the host. Various substrates including extracellular matrix proteins, growth factors, and cytokines are cleaved by meprins. Meprin β on the apical side of intestinal epithelial cells detaches the constitutive mucus due to the prevention of bacterial overgrowth (41). Meprin α deficient mice were more susceptible to dextran sulfate sodium-induced experimental colitis and underwent larger colon disorders and inflammation than wild-type mice (42). Meprins may function as a mucosal defense mechanism that maintains the intestinal epithelium against potential toxic peptides and also against enteric commensal and pathogenic bacteria by regulating the interaction between microbes and the host mucosa (42). Another primary protein neighbor of ACE2; namely Dipeptidyl peptidase 4 (DPP4) (also known as cluster of differentiation 26 (CD26)), is a transmembrane glycoprotein presented ubiquitously in several tissues such as lung, kidney, liver, gut, and immune cells (43). DPP4 as a serine exo-peptidase is involved in various physiological processes and can cleave a wide range of substrates including growth factors, chemokines, neuropeptides, and vasoactive peptides. DPP4 modulates immune responses by functioning as a costimulatory molecule on T-cells and regulates glucose homeostasis by degrading incretin hormones (43). The DPP-4 activity, as one of the peptidases at the surface of enterocytes, participates in the last step of protein digestion (44). According to recent studies, it was suggested that SARS–CoV-2, like MERS–Co-V, may use the DPP4/CD26 receptor as a co-receptor when virus enters the cells via ACE2. The co-expression of ACE2 and DPP4/CD26, as receptors of S glycoproteins, could hypothesize that verity human CoVs target similar cell types across various human tissues and explain the emergence of similar clinical characteristics in patients infected with verity CoVs (45). In our results, ghrelin (GHRL) as a primary protein neighbor of ACE2 links together two of the detected highly clustered modules. Ghrelin is a peptide hormone mostly secreted by X/A-like gastric cells, functions through the growth hormone secretagogue receptor (GHSR), and exhibits a modulatory role in the immune system (46). Ghrelin also induces gastric motility and emptying as well as motility in the small and large intestine. Ghrelin has been shown to be affected in multiple gastrointestinal diseases and disorders such as inflammatory bowel disease, coeliac disease, infectious diseases, functional disorders and diabetes gastroenteropathy. This demonstrates that ghrelin is implicated in the pathophysiology of gastrointestinal diseases and disorders (47). 

In this study, we also enriched HNF proteins (HNF4A, HNF1A, and HNF1B) as transcription factors which regulate expression of the ACE2 target gene. HNF4 factors were identified in one recent study as key transcriptional regulators of SARS-CoV-2 entry genes in the intestine (48). Chen et al., using epigenomic approaches and mouse genetic models, reported that HNF4 factors bind to the loci of the ACE2 gene, alter chromatin looping, shape epigenetic modifications, and ultimately show a dramatic impact on its expression upon transcription factor knockout (48). Our predictions, for HNF1A and HNF1B factors, have been formerly reported experimentally to drive ACE2 expression in pancreatic islet cells and insulinoma cells, respectively (49). Recently, Barker et al., using bioinformatics tools, also predicted that the HNF1A transcription factor in colon tissue has high positive correlation with the expression of ACE2 (50). CDX2 was found to be another important transcription factor that was enriched in this study. It was shown that this factor regulates furin expression during intestinal epithelial cell differentiation (51). The small intestine is rich in furin, a serine protease, that can cleave the S protein of the coronavirus into two “pinchers” (S1 and 2). The cleaving of the S protein into S1 and S2 is important for the binding of the virion to both the ACE2 receptor and the cell membrane (39). Indeed, furin is a broadly distributed enzyme in the small intestine and is the main enzyme in the process of activation of other enteric toxins (39).

Moreover, in this study, we extracted a list of 60 drugs that interact with the 176 gene list. Some of these reported drugs can be beneficial in use against COVID-19 infection.

One preliminary clinical study reported that treatment with renin inhibitor aliskiren, as an antihypertensive agent, is effective and safe for severe COVID-19 patients complicated with hypertension (52). In another study, it was reported that aliskiren, by molecular docking, showed higher energies of binding than that of the co-crystallized ligand N3 with COVID-19 main protease M^pro ^(53). They suggested that aliskiren can be a repurposing drug for the treatment of COVID-19. Gliptins are antidiabetic drugs controlling glucose homeostasis by the prevention of DPP4 enzymatic activity. Gliptins can protect endothelial function by their reported anti-inflammatory, anti-oxidant, and potentially protective effects on the vascular system, which are beneficial aspects in the fight against COVID-19 (54). Gliptins might be also used to restrain SARS-CoV-2 binding to host cells. DPP4 inhibitor ability to inhibit coronavirus entry into host cells has also been investigated (54). Another noteworthy point is that a recent study reported that anagliptin might exert a cholesterol-lowering action through DPP4-dependent and glucagon-like peptide 1-independent suppression of intestinal cholesterol transport (55).

Candesartan, as an angiotensin receptor blocker (ARB), reduces inflammation and protects lung and brain functions, and it has recently been reported that candesartan can be therapeutically effective in COVID-19 patients and ameliorate the cytokine storm (56). One recent first-time study demonstrated that candesartan treatment alleviates hypertension-associated pathophysiological alterations in the gut, enhances microbial production of short-chain fatty acids, and protects gut *Lactobacillus* under hypertensive conditions. This information sheds novel light on the pharmacological implications of candesartan (57). Other ARBs such as losartan and telmisartan were enriched in our study as therapeutics for SARS-CoV-2 virus infections. These drugs are widely used in the clinic since the 1990s for control of hypertension and kidney disorders and are known as safe drugs that are rarely implicated in adverse drug events (58). Telmisartan, which is well absorbed after oral administration, has the longest plasma half-life (24 hr), and achieves the highest tissue concentrations because of its high lipid solubility and high levels of dissemination (500 L). This drug separates more slowly after attachment to the Ang receptor I, leading to an apparently irreversible block (59). Recently, a randomized open-label controlled trial began enrollment of patients in Hospital de Clínicas José de San Martín (School of Medicine, University of Buenos Aires, Argentina). Clinical studies to investigate the safety of Telmisartan in healthy cases, or in hypertensive patients receiving daily doses of up to 160 mg, indicated no distinction among those treated with Telmisartan and the placebo group in frequency and severity of detrimental effects (59). Another recent study reported that Telmisartan can attenuate colon inflammation, oxidative perturbations, and apoptosis in a rat model with experimental inflammatory bowel disease (60). Valsartan is another ARB that was enriched in this study. The PRAETORIAN-COVID trial has been begun as a project to provide the much-expected evidence regarding the controversy on the acts of Valsartan in patients with SARS-CoV-2 infectious disease (61). 

Carvedilol is a drug with vasodilating properties initially designed for managing hypertension and coronary artery disease. Unlike ACE inhibitors that enhance the expression of ACE2, carvedilol reduces its expression, therefore, this drug can be useful for all COVID-19 patients (62). Carvedilol can fight COVID-19 through another mechanism because it has interleukin 6 (IL-6) suppressing properties and which plays a major role in the inflammatory cascade of COVID-19 (62).

Gamma-linolenic acid, as a bioactive lipid, inhibits the production of pro-inflammatory IL-6 and TNF-α and could be employed to treat cytokine storm that is seen in COVID-19 patients. Reports show that gamma-linolenic acid inactivates enveloped viruses including COVID-19, thus, infusions of appropriate amounts of this acid are of significant therapeutic benefit in treating COVID-19 (63). According to these data, a prospective double-blinded controlled 14-day trial on 30 SARS-CoV-2 positive cases has been conducted (Anti-inflammatory/Antioxidant Oral Nutrition Supplementation in COVID-19 [ONSCOVID19]; NCT04323228). The participants were randomly assigned to two groups (n = 15/each); intervention (IG) and placebo (PG). The IG group received an anti-inflammatory and antioxidant oral nutrition supplement (ONS) enriched in eicosapentaenoic acid, gamma-linolenic acid, and antioxidants daily, while the PG group received an isocaloric placebo. It can be concluded that with more maintenance of the nutritional status of infected patients the anti-inflammatory-antioxidant ONS might contribute to the decrease of COVID-19 severity. 

Studies have shown that Verapamil can interfere with coronavirus entry and amplification by blocking ion channels. It was proven that Verapamil is effective against RNA viruses in vitro and inhibited filovirus infection in cell cultures and mouse models (64). Interestingly, a randomized trial has been commenced comparing Verapamil and amiodarone with usual care in hospitalized patients with confirmed COVID-19 (Amiodarone or Verapamil in COVID-19 Hospitalized Patients with Symptoms [ReCOVery-SIRIO]; NCT04351763). 

The human protein-host protein interactome of SARS-CoV-2 infection in the small intestine was investigated. The outcomes indicate that ACE2 and its interacting proteins in the small intestine may explain gastrointestinal symptoms and intestinal inflammation. The results suggest that antiviral targeting these interactions may improve the condition of COVID-19 patients; however, more research is needed to find possible mechanisms of the gastrointestinal symptoms and confirm associated biomarkers..

## References

[1] Cao W (2020). Clinical features and laboratory inspection of novel coronavirus pneumonia (COVID-19) in Xiangyang, Hubei. medRxiv.

[2] Sohrabi C, Alsafi Z, O’Neill N, Khan M, Kerwan A, Al-Jabir A (2020). World Health Organization declares global emergency: A review of the 2019 novel coronavirus (COVID-19). Int J Surg.

[3] Li Q, Guan X, Wu P, Wang X, Zhou L, Tong Y (2020). Early transmission dynamics in Wuhan, China, of novel coronavirus–infected pneumonia. N Engl J Med.

[4] Huang C, Wang Y, Li X, Ren L, Zhao J, Hu Y (2020). Clinical features of patients infected with 2019 novel coronavirus in Wuhan, China. The lancet.

[5] Fang Z, Yi F, Wu K, Lai K, Sun X, Zhong N (2020). Clinical characteristics of 2019 coronavirus pneumonia (COVID-19): an updated systematic review. MedRxiv.

[6] Chen N, Zhou M, Dong X, Qu J, Gong F, Han Y (2020). Epidemiological and clinical characteristics of 99 cases of 2019 novel coronavirus pneumonia in Wuhan, China: a descriptive study. The Lancet.

[7] Xu X, Yu C, Qu J, Zhang L, Jiang S, Huang D (2020). Imaging and clinical features of patients with 2019 novel coronavirus SARS-CoV-2. Eur J Nucl Med Mol Imaging.

[8] Chan JFW, Yuan S, Kok KH, To KKW, Chu H, Yang J (2020). A familial cluster of pneumonia associated with the 2019 novel coronavirus indicating person-to-person transmission: a study of a family cluster. The Lancet.

[9] Zhou P, Yang XL, Wang XG, Hu B, Zhang L, Zhang W (2020). A pneumonia outbreak associated with a new coronavirus of probable bat origin. Nature.

[10] Hamming I, Timens W, Bulthuis M, Lely A, Navis Gv, van Goor H (2004). Tissue distribution of ACE2 protein, the functional receptor for SARS coronavirus A first step in understanding SARS pathogenesis. J Pathol.

[11] Nikhra V (2020). The Agent and Host Factors in Covid-19: Exploring Pathogenesis and Therapeutic Implications. Biomed J Sci Techn Res.

[12] Kuba K, Imai Y, Rao S, Gao H, Guo F, Guan B (2005). A crucial role of angiotensin converting enzyme 2 (ACE2) in SARS coronavirus–induced lung injury. Nature Med.

[13] Hashimoto T, Perlot T, Rehman A, Trichereau J, Ishiguro H, Paolino M (2012). ACE2 links amino acid malnutrition to microbial ecology and intestinal inflammation. Nature.

[14] Li Z, Bai T, Yang L, Hou X (2020). Discovery of potential drugs for COVID-19 based on the connectivity map.

[15] Feng Z, Wang Y, Qi W (2020). The small intestine, an underestimated site of SARS-CoV-2 infection: from red queen effect to probiotics. Preprints.

[16] Liang W, Feng Z, Rao S, Xiao C, Xue X, Lin Z (2020). Diarrhoea may be underestimated: a missing link in 2019 novel coronavirus. Gut.

[17] Zhang H, Li HB, Lyu JR, Lei XM, Li W, Wu G (2020). Specific ACE2 expression in small intestinal enterocytes may cause gastrointestinal symptoms and injury after 2019-nCoV infection. Int J Infect Dis.

[18] Liu X, Yu X, Zack DJ, Zhu H, Qian J (2008). TiGER: a database for tissue-specific gene expression and regulation. BMC Bioinf.

[19] Gheblawi M, Wang K, Viveiros A, Nguyen Q, Zhong JC, Turner AJ (2020). Angiotensin-converting enzyme 2: SARS-CoV-2 receptor and regulator of the renin-angiotensin system: celebrating the 20th anniversary of the discovery of ACE2. Circul Res.

[20] Wicik Z, Eyileten C, Jakubik D, Pavao R, Siller-Matula JM, Postula M (2020). ACE2 interaction networks in COVID-19: a physiological framework for prediction of outcome in patients with cardiovascular risk factors. BioRxiv..

[21] Karunakaran KB, Balakrishnan N, Ganapathiraju MK (2020). Interactome of SARS-CoV-2/nCoV19 modulated host proteins with computationally predicted PPIs.

[22] Consortium U (2019). UniProt: a worldwide hub of protein knowledge. Nucleic Acids Res.

[23] Stelzer G, Rosen N, Plaschkes I, Zimmerman S, Twik M, Fishilevich S (2016). The GeneCards suite: from gene data mining to disease genome sequence analyses. Curr Protoc Bioinf.

[24] Szklarczyk D, Gable AL, Lyon D, Junge A, Wyder S, Huerta-Cepas J (2019). STRING v11: protein–protein association networks with increased coverage, supporting functional discovery in genome-wide experimental datasets. Nucleic Acids Res.

[25] Hoffmann M, Kleine-Weber H, Schroeder S, Krüger N, Herrler T, Erichsen S (2020). SARS-CoV-2 cell entry depends on ACE2 and TMPRSS2 and is blocked by a clinically proven protease inhibitor. Cell.

[26] Gordon D, Jang G, Bouhaddou M, Xu J, Obernier K, O’Meara M (2020). A SARS-CoV-2-Human Protein-Protein Interaction Map Reveals Drug Targets and Potential Drug Repurposing. Nature.

[27] Kumar R, Verma H, Singhvi N, Sood U, Gupta V, Singh M (2020). Comparative Genomic Analysis of Rapidly Evolving SARS-CoV-2 Reveals Mosaic Pattern of Phylogeographical Distribution. Msystems.

[28] Dennis G, Sherman BT, Hosack DA, Yang J, Gao W, Lane HC (2003). DAVID: database for annotation, visualization, and integrated discovery. Genome Biol.

[29] Shannon P, Markiel A, Ozier O, Baliga NS, Wang JT, Ramage D (2003). Cytoscape: a software environment for integrated models of biomolecular interaction networks. Genome Res.

[30] Bader GD, Hogue CW (2003). An automated method for finding molecular complexes in large protein interaction networks. BMC Bioinf.

[31] Kanehisa M, Furumichi M, Tanabe M, Sato Y, Morishima K (2017). KEGG: new perspectives on genomes, pathways, diseases and drugs. Nucleic Acids Res.

[32] Stöckel D, Kehl T, Trampert P, Schneider L, Backes C, Ludwig N (2016). Multi-omics enrichment analysis using the GeneTrail2 web service. Bioinformatics.

[33] Matys V, Fricke E, Geffers R, Gößling E, Haubrock M, Hehl R (2003). TRANSFAC®: transcriptional regulation, from patterns to profiles. Nucleic Acids Res.

[34] Becker KG, Barnes KC, Bright TJ, Wang SA (2004). The genetic association database. Nat Genet.

[35] Brown AS, Patel CJ (2017). A standard database for drug repositioning. Sci Data.

[36] Szklarczyk D, Santos A, von Mering C, Jensen LJ, Bork P, Kuhn M (2016). STITCH 5: augmenting protein–chemical interaction networks with tissue and affinity data. Nucleic Acids Res.

[37] Wu J, Deng W, Li S, Yang X (2020). Advances in research on ACE2 as a receptor for 2019-nCoV. Cell Mol Life Sci.

[38] Zhang H, Penninger JM, Li Y, Zhong N, Slutsky AS (2020). Angiotensin-converting enzyme 2 (ACE2) as a SARS-CoV-2 receptor: molecular mechanisms and potential therapeutic target. Intensive Care Med.

[39] Mönkemüller K, Fry L, Rickes S (2020). Covid-19, Coronavirus, SARS-CoV-2 and the small bowel. Rev Esp Enferm Dig.

[40] Camargo SM, Singer D, Makrides V, Huggel K, Pos KM, Wagner CA (2009). Tissue-specific amino acid transporter partners ACE2 and collectrin differentially interact with hartnup mutations. Gastroenterology.

[41] Arnold P, Otte A, Becker-Pauly C (2017). Meprin metalloproteases: molecular regulation and function in inflammation and fibrosis. Biochim Biophys Acta Mol Cell Res.

[42] Vazeille E, Bringer MA, Gardarin A, Chambon C, Becker-Pauly C, Pender SL (2011). Role of meprins to protect ileal mucosa of Crohn's disease patients from colonization by adherent-invasive E. coli. PLoS One.

[43] Strollo R, Pozzilli P (2020). DPP4 inhibition: preventing SARS‐CoV‐2 infection and/or progression of COVID‐19?. Diabetes Metab Res Rev.

[44] Olivares M, Schüppel V, Hassan AM, Beaumont M, Neyrinck AM, Bindels LB (2018). The potential role of the dipeptidyl peptidase-4-like activity from the gut microbiota on the host health. Front Microbiol.

[45] Solerte SB, Di Sabatino A, Galli M, Fiorina P (2020). Dipeptidyl peptidase-4 (DPP4) inhibition in COVID-19. Acta Diabetol.

[46] Pereira JAdS, Silva FCd, de Moraes-Vieira PMM (2017). The impact of ghrelin in metabolic diseases: an immune perspective. J Diabetes Res.

[47] El-Salhy M (2009). Ghrelin in gastrointestinal diseases and disorders: a possible role in the pathophysiology and clinical implications. Int J Mol Med.

[48] Chen L, Marishta A, Ellison CE, Verzi MP (2020). Identification of transcription factors regulating SARS-CoV-2 entry genes in the intestine. Cell Mol Gastroenterol Hepatol.

[49] Pedersen KB, Chhabra KH, Nguyen VK, Xia H, Lazartigues E (2013). The transcription factor HNF1α induces expression of angiotensin-converting enzyme 2 (ACE2) in pancreatic islets from evolutionarily conserved promoter motifs. Biochim Biophys Acta Mol Cell Res.

[50] Barker H, Parkkila S (2020). Bioinformatic characterization of angiotensin-converting enzyme 2, the entry receptor for SARS-CoV-2. BioRxiv..

[51] Gendron F-P, Mongrain S, Laprise P, McMahon S, Dubois CM, Blais M (2006). The CDX2 transcription factor regulates furin expression during intestinal epithelial cell differentiation. Am J Physiol Gastrointest Liver Physiol.

[52] Guo Y, Zeng J, Li Q, Li P, Luo F, Zhang W (2020). Preliminary clinical study of direct renin inhibitor aliskiren in the treatment of severe COVID-19 patients with hypertension. Zhonghua Nei Ke Za Zhi.

[53] Aly OM (2020). Molecular docking reveals the potential of aliskiren, dipyridamole, mopidamol, rosuvastatin, rolitetracycline and metamizole to inhibit COVID-19 virus main protease. chemrxiv.

[54] Valencia I, Peiró C, Lorenzo Ó, Sánchez-Ferrer CF, Eckel J, Romacho T (2020). DPP4 and ACE2 in diabetes and COVID-19: therapeutic targets for cardiovascular complications?. Front Pharmacol.

[55] Goto M, Furuta S, Yamashita S, Hashimoto H, Yano W, Inoue N (2018). Dipeptidyl peptidase 4 inhibitor anagliptin ameliorates hypercholesterolemia in hypercholesterolemic mice through inhibition of intestinal cholesterol transport. J Diabetes Invest.

[56] Elkahloun AG, Saavedra JM (2020). Candesartan could ameliorate the COVID-19 cytokine storm. Biomed Pharmacother.

[57] Wu D, Tang X, Ding L, Cui J, Wang P, Du X (2019). Candesartan attenuates hypertension-associated pathophysiological alterations in the gut. Biomed Pharmacother.

[58] Gurwitz D (2020). Angiotensin receptor blockers as tentative SARS‐CoV‐2 therapeutics. Drug Dev Res.

[59] Rothlin RP, Vetulli HM, Duarte M, Pelorosso FG (2020). Telmisartan as tentative angiotensin receptor blocker therapeutic for COVID‐19. Drug Dev Res.

[60] Arab HH, Al-Shorbagy MY, Abdallah DM, Nassar NN (2014). Telmisartan attenuates colon inflammation, oxidative perturbations and apoptosis in a rat model of experimental inflammatory bowel disease. PLoS One.

[61] Gommans DF, Nas J, Pinto-Sietsma SJ, Koop Y, Konst RE, Mensink F (2020). Rationale and design of the PRAETORIAN-COVID trial: A double-blind, placebo-controlled randomized clinical trial with valsartan for PRevention of Acute rEspiraTORy dIstress syndrome in hospitAlized patieNts with SARS-COV-2 Infection Disease. Am Heart J.

[62] Skayem C, Ayoub N (2020). Carvedilol and COVID-19: A Potential Role in Reducing Infectivity and Infection Severity. Am J Med Sci.

[63] Das UN (2020). Bioactive Lipids as Mediators of the Beneficial Action (s) of Mesenchymal Stem Cells in COVID-19. Aging Dis.

[64] Castaldo N, Aimo A, Castiglione V, Padalino C, Emdin M, Tascini C (2020). Safety and efficacy of amiodarone in a patient with COVID-19. JACC Case Rep.

